# Tissue engineering in age-related macular degeneration: a mini-review

**DOI:** 10.1186/s13036-022-00291-y

**Published:** 2022-05-16

**Authors:** Andres Wu, Renhao Lu, Esak Lee

**Affiliations:** 1 Nancy E. and Peter C. Meinig School of Biomedical Engineering, Cornell University, Ithaca, NY 14853 USA; 2 Ann S. Bowers College of Computing and Information Science, Cornell University, Ithaca, NY 14853 USA

**Keywords:** Age-related macular degeneration (AMD), Tissue-engineered models, Microfluidic devices, 3D cell culture, 2D cell culture, Non-exudative (‘dry) AMD, Exudative (‘wet) AMD, Retinal pigment epithelium, Bruchs membrane, Drusen, Macular neovascularization, Macular atrophy

## Abstract

Age-related macular degeneration (AMD) is a progressive, degenerative disease of the macula, leading to severe visual loss in the elderly population. There are two types of AMD: non-exudative (‘dry’) AMD and exudative (‘wet’) AMD. Non-exudative AMD is characterized by drusen formation and macular atrophy, while the blood vessels are not leaky. Exudative AMD is a more advanced form of the disease, featured with abnormal blood vessel growth and vascular leakage. Even though anti-angiogenic therapies have been effective in treating wet AMD by normalizing blood vessels, there is no treatment available to prevent or treat dry AMD. Currently, the mechanisms of drusen formation and macular atrophy in the dry AMD are poorly understood, in part because the currently available in vivo models of AMD could not decouple and isolate the complex biological and biophysical factors in the macular region for a detailed mechanism study, including the complement system, angiogenesis factors, extracellular matrix, etc. In the present review article, we describe the biological background of AMD and the key cells and structures in AMD, including retinal epithelium, photoreceptor, Bruch’s membrane, and choriocapillaris. We also discuss pre-clinical animal models of AMD and in vivo tissue-engineered approaches, including cell suspension injection and organoid-derived cell sheet transplantation. We also discuss in vitro tissue-engineered models for AMD research. Specifically, we evaluate and compare currently available two- and three-dimensional AMD tissue-engineered models that mimic key anatomical players in AMD progression, including pathophysiological characteristics in Bruch’s membrane, photoreceptor, and choriocapillaris. Finally, we discuss the limitation of current AMD models and future directions.

## Background

Age-related macular degeneration (AMD) is a progressive, degenerative disease of the macula, a small area at the center of the retina [1]. It is in adults older than 50 years in industrialized countries and its incidence has been rising as the life expectancy of the global population has increased [2]. There are two types of AMD: non-exudative (‘dry’) AMD and exudative (‘wet’) AMD. Non-exudative AMD and exudative AMD account for 90% and 10% patients diagnosed with AMD, respectively [3]. The clinical hallmark of non-exudative AMD is drusen, yellow deposits made up of lipoproteins, found in between the retinal pigment epithelium (RPE) and Bruch’s membrane (BrM) [4]. This non-exudative AMD is divided into early, intermediate, and late stages based on the size and the degree of drusen deposition. The early stage is recognized by having multiple small (< 63 µm) or more than one intermediate drusen (63 µm ~ 125 µm); the intermediate stage is characterized by many intermediate or one large drusen (> 125 µm); the late stage is characterized by geographic atrophy (GA) involves degeneration of the RPE, retina and the choriocapillaris with well-demarcated borders, resembling the map of a ‘continent’. Wet AMD is characterized by vascular growth from the choroid penetrating Bruch’s membrane, referred to as macular neovascularization (MNV), which accounts for 10% of total AMD patients [5]. The newly formed blood vessels leak fluid, causing visual impairment and blindness, which is mediated by drusen-mediated activation of vascular endothelial growth factor (VEGF) expression in the lesion [6]. Thus, anti-angiogenic therapies using anti-VEGF agents in the past 15 years have significantly reduced the blindness rates for those wet AMD patients with MNV [7]. Even though anti-VEGF is effective in exudative AMD, there is no treatment available to prevent or treat non-exudative (or ‘dry’) AMD initiated by drusen formation and leading to MA. Thus, the drusen formation and MA remain an area of significant unmet medical need [8].

We acknowledge that animal models have been widely used to study ocular biology and disease such as AMD [9]. Despite the contributions of the animal models, it is often difficult to isolate the relative contributions of complex biological factors and biophysical cues in the animal models, including inflammatory cytokines, reactive oxidative species (ROS), interstitial pressure, etc., thus these in vivo models are often not as helpful in identifying detailed mechanisms underlying multifactorial AMD pathogenesis [10]. By contrast, traditional cell cultures in two-dimensional (2D) dishes or transwell permit such identification, as they are a highly controllable model system, but they do not recapitulate the three-dimensional (3D) in vivo organization of these ocular tissues [11–13]. Therefore, there has been a clear, unmet need for 3D culture that reconstitutes human ocular structure to permit controlled experiments investigating drusen formation, deposition, and macular atrophy in AMD [9]. The purpose of the current review is to provide an update on the in vitro bioengineered methods to study AMD.

### Main text

#### Basic biology of age-related macular degeneration (AMD)

**Fig. 1:**
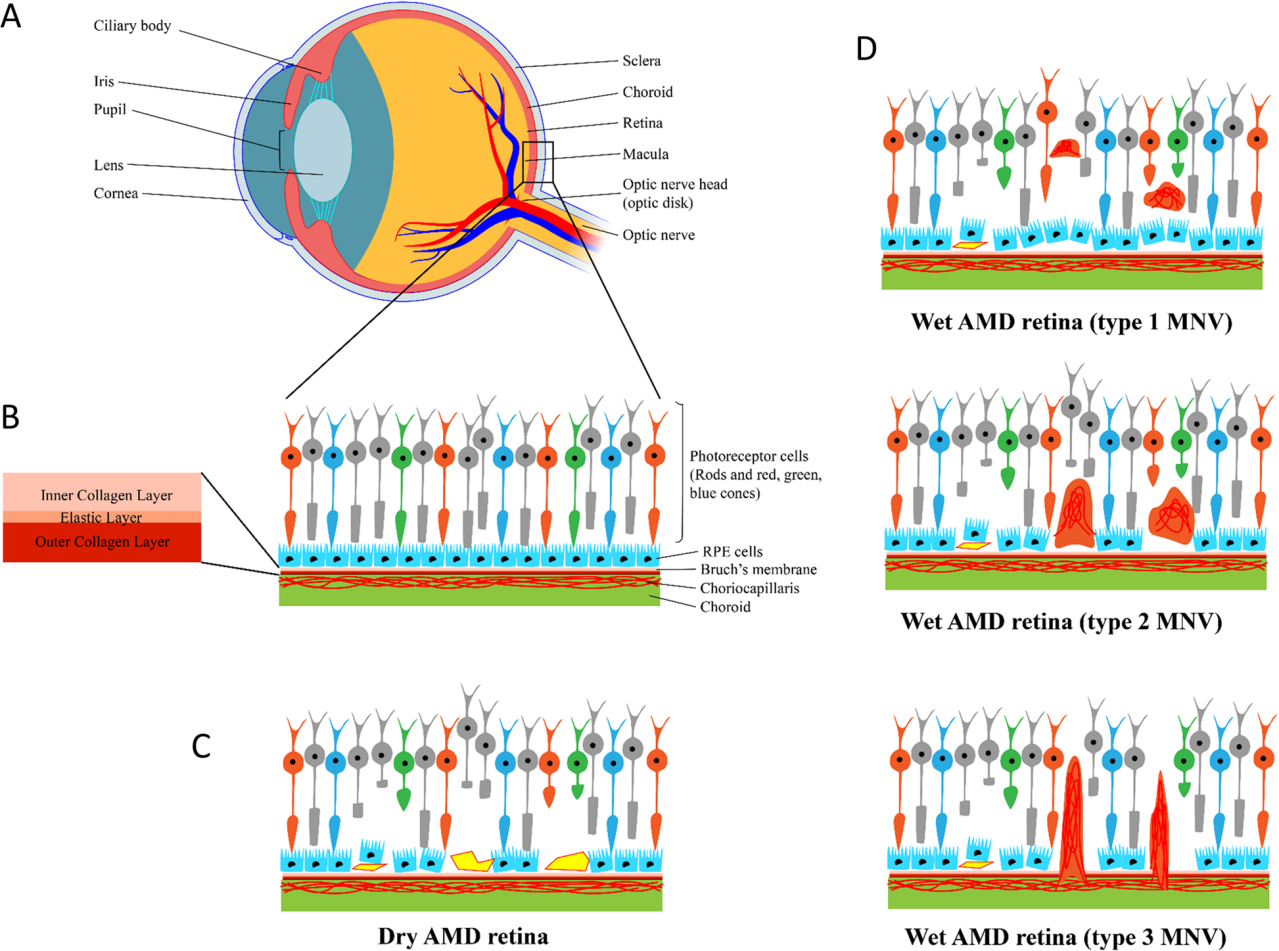
Eye structure and AMD pathology. **A** Physiology structure of an entire eye. **B** Enlarged description of macular and Bruch’s membrane structure. **C** Pathology of dry AMD, including drusen deposition (yellow) and RPE elevation. **D** Pathology of 3 types of wet AMD. Type 1 macular neovascularization (MNV) is an ingrowth of vessels without outer retinal disruption. Type 2 MNV is the proliferation of new vessels with outer retinal disruption. Type 3 MNV is a downgrowth of vessels from the retinal vascular plexus toward the outer retina [22]

The retina is one of the highest oxygen consuming tissues in the body, making it one of the most metabolically active tissues (Fig. 1A). It therefore requires an efficient mechanism to maintain homeostasis as well as meet metabolic needs of the retinal neurons [14]. There are three major cellular types including vascular cells, glial cells, and neurons that comprise the retina. Similar to the central nervous system, these cells are intimately interacting to each other in the retina to form a functional unit called the retinal neurovascular unit (NVU) whose function is to deliver the metabolic fuels required for vision in a timely manner, and remove the metabolic wastes generated during the metabolism of vision [15]. The retinal NVU is composed of the photoreceptors, Bruch's membrane (BrM), the retinal pigment epithelium (RPE), choriocapillaris (CC), and deep capillary plexus (Fig. 1B) [16]. The photoreceptors are the group of cells that are converting light into biological signals that are sent to the brain. Because positively charged ions consistently enter unstimulated photoreceptors to keep dark the photoceptor disks continuously, retina is one of the most metabolically active parts in the human body [17]. The choriocapillaris (CC) is a fenestrated layer of capillaries that provides nutrients and removes waste products from the photoreceptors and the RPE. Bruch's membrane (BrM) is an extracellular matrix that lies between the CC and the RPE, which functionally serves as a biological barrier for transport of the nutrients and metabolites [18]. It consists of an inner collagenous layer (adjacent to the RPE), a middle elastic layer, and an outer collagenous layer (adjacent to the CC). The retinal pigment epithelium (RPE) is a monolayer of polarized hexagonal cells whose apices have multiple villous processes that are intimately related to the photoreceptor outer segment. By phagocytosing the outer segment discs that are shed daily, the RPE plays a critical role in retinoid recycling during phototransduction [19]. Finally, the lateral intercellular junctional complexes that join contiguous RPE cells form the outer blood retinal barrier [20, 21].

The AMD is a complex, chronic, multifactorial disease characterized by an aging macula in individuals with genetic mutations and environmental risk factors whose pathophysiology is poorly understood [23]. It appears that genetic mutations, particularly in the complement genes, play an important role in establishing the disease.A large genome-wide association study identified 34 loci that clustered in three main pathways in AMD progression (in ascending order of *p*-value): the complement system, extracellular matrix remodeling, and lipoprotein metabolism [24]. Risk factors of AMD include aging, smoking, higher body mass index, cardiovascular disease, high fat diet, etc., however, aging is the most consistent risk factor among them [25]. Aging causes choroidal thinning which is most likely due to a decreased choroidal vascular volume, secondary to the resultant decrease in choroidal blood perfusion. The severity of the choroidal perfusion deficits, particularly in the central macula, has been correlated with AMD severity and progression [26–28]. These changes in choroidal hemodynamics appear to play a crucial role in the progression of intermediate AMD to late AMD [29]. Meanwhile, aging also influences the transport function of BrM, by thickening and cross-linking the barrier. As the process goes on, the lipoproteins secreted from functional RPE cells would not be able to transport through the BrM and choroidal blood endothelium, causing the impaired egress [30]. Hence, the BrM accumulates with lipids, especially lipoproteins that contain apolipoprotein B and E, cholesterol and 7-ketocholesterol. As a result, the aging macula experiences the deposition of lipid rich extracellular deposits known as drusen and subretinal drusenoid deposits (SDD), causing dry AMD (Fig. 1C) [31]. The lipoprotein deposited between the RPE and BrM (the subRPE space) is called Drusen, and the deposition between the RPE and the photoreceptors (the subretinal space) is called SDD [18, 30, 32]. These deposits trigger the complement cascade, recruit the macrophage through the breaching BrM into sub RPE space, and activate the microglia in subretinal space, which further leads to local inflammatory responses. The complement system, a part of the innate immune system is unspecific, thus can lead to macular neovascularization (wet AMD) and macular atrophy (Fig. 1D). Patients with genetic polymorphisms of the complement genes are unable to turn off the complement cascade in the disease progression [33, 34].

### Preclinical animal models and tissue-engineered approaches for in Vivo transplantation

In the past, there was a strong reliance on animal models to further our understanding of the mechanisms of AMD and to test new therapies. A major limitation in translating observations gained from animal models to human disease is the differences between the anatomy of these animals and humans [35]. For instance, the rodents, such as mice and rats, has a similar cone density in entire retina, hence don’t have anatomical macula [36]. Non-human primates are the only animals that has macular structure that is similar to human, showed spontaneously develop drusenoid lesions. However, non-human primates haven’t showed advanced AMD hallmarks, such as MA or MNV [37, 38]. Meanwhile, these are costly to maintain, difficult to manipulate genetically, and manifest a slow time course of disease progression compared with rodent models [39]. Despite the absence of macula, rodent models have been able to recreate several of the histological features of dry AMD including drusen-like extracellular deposits between RPE and BrM, choroid neovascularization, and subretinal microglia accumulation [40]. Notably, the microglia accumulation was not found in human eyes with AMD, suggesting another distinction between human and rodent model [41]. The laser-induced macular neovascularization (MNV) in both rodents and non-human primates have served as important models to study drug treatments in exudative AMD [42–44]. The optimal rodent model differs depending on which stage or type of AMD phenotype one wants to model. Although several animal models of AMD have been developed, none of them has been able to demonstrate the full human AMD spectrum [40]. Therefore, a lack of adequate animal models has hampered progress in our understanding of the mechanisms that lead to macular atrophy.

Prior to the introduction of pharmacological therapy with anti-VEGF drugs, surgical approaches were tried in eyes with exudative AMD. One example is macular translocation, which is a surgical procedure that moves the macular neuroretina to the area of RPE without neovascularization [45]. Although most eyes that underwent the macular translocation did not recover vision, some eyes experienced functional improvement once the macular photoreceptors were coupled to a bed of healthy RPE cells. This study, together with the fact that the RPE can heal after surgical removal of neovascular membranes, has served as a proof of concept that reconstitution of the macular function was possible by transplanting healthy RPE cell bed [46]. Transplantation of sheets of homologous fetal RPE, suspensions of autologous iris pigment epithelial cells or RPE cells have been tried but with little functional success [47, 48]. In contrast, an autologous free RPE-choroid graft was able to improve the visual acuity in eyes with wet AMD. However, serious intra-operative and post-operative complications, including retinal detachment, proliferative vitreoretinopathy, and recurrence of the MNV, were reported as side effects of these surgical procedures [49].

**Fig. 2:**
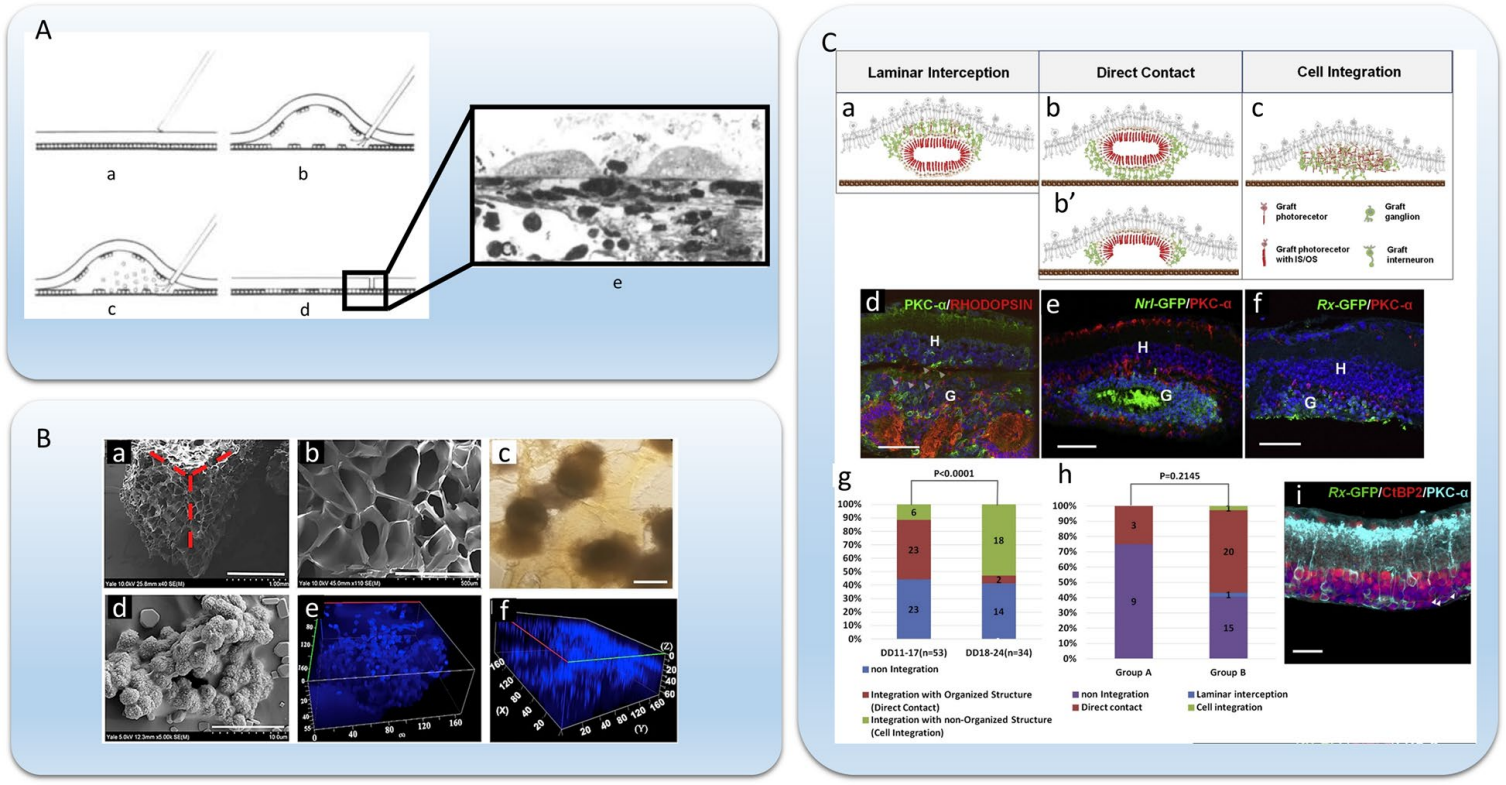
Tissue-Engineered Approaches for In vivo Transplantation.** A** A schematic illustration of transplantation of RPE cell suspension into neural rerina. (a) Micropipette containing cell suspension is positioned adjacent to neural retina. (b) Small amount of buffered salt solution (BSS) at tip of the pipette is slowly injected through retina into subretinal space. As bleb detachment forms, patches of host RPE cells lift off with neural retina, creating areas of bare Bruch's membrane. (c) With continued injection, cell suspension enters subretinal space. (d) Reattachment of retina occurs within 24 to 48 h of the RPE cell transplantation. (e) SEM image of two attached RPE cells onto the bare BrM surface one hour after injection [50]. **B** Biodegradable 3D gelatin, chondroitin sulfate, and hyaluronic acid (GCH) scaffold for retina cell differentiation and transplant. (a) Scanning electron micrograph shows three faces of a block of scaffold. (b) A higher magnification of one face. Light micrograph (c) and scanning electron micrograph (d) show embryoid bodies on the scaffold one-day post-seeding. (e) One-week post-seeding, cultures were stained with DAPI (blue) to reveal cell nuclei. Three-dimensional reconstruction from confocal micrographs demonstrated that cells migrated the thickness of the scaffold. (f) After three weeks, cells homogenously populated most of the scaffold, but acellular areas were present [52]. **C** Transplantation of organoid-derived retina-like sheets with rd1 host retina. Schematic diagrams show three typical patterns of integration with rd1 host retina of the transplanted grafts. (a) Pattern 1: laminar interception. Graft INL was present between host INL and graft ONL. (b) Pattern 2: direct contact. The graft ONL was adjacent to the host INL. (c) Pattern 3: cell integration. The graft ONL structure was disorganized, similar to what was observed for cell transplantation. (d) A typical image of pattern 1. RHODOPSIN + photoreceptors from DD16 *Nrl*-GFP miPSC-derived retinal sheets migrate toward the host retina (white arrowhead). H, host; G, graft. (e) A typical image of pattern 2. DD16 *Nrl*-GFP miPSC-derived retinal sheets show structured ONL directly contacting host INL. H, host; G, graft. (f) A typical image of pattern 3. DD18 Rx-GFP mESC-derived retinal grafts show disorganized patterns similar to those observed for cell transplantation. H, host; G, graft. Scale bars, (**B**) a, 1000 μm; b&c, 500 μm; d, 100 μm; e&f, scale in μm. (**C**) d-f, 50 μm (D–F); I, 20 μm. Figures were adapted with permission from [51–53]

Ever since the observation in 1987 that transplanted rabbit RPE cells into the subretinal space are able to survive and phagocytose the photoreceptor outer segments [50], several groups have banked on cell therapy as a promising treatment alternative for AMD. RPE cell suspension was positioned adjacent to neural retina. Before injecting RPE cells, a buffer solution was slowly injected through retina into subretinal space to form bleb detachment. With continued RPE cell injection, cell suspension enters subretinal space [51] (Fig. 2A). While retinal allografts in pigs and primates are associated with a significant immune response, an autologous source of cells is preferred to avoid immunosuppressive drugs that are expensive, toxic, and life-long. Cells derived from the patient for whom they are intended would be the best possible immunologic match.

The main autologous cell sources for retinal implants include embryonic or fetal derived stem cells, adult tissue derived stem cells and induced pluripotent stem cells (iPSCs) [35]. The plasticity and unlimited capacity for self-renewal of embryonic stem cells need to be balanced against the potential safety issues including tumorigenesis, potential immune rejection, possible ethical issues, and the risk of differentiating into unwanted cell types. Adult tissues derived those stem cells may serve as a source of autologous multipotent cells but are not easy to come by and may contain the genetic cause of the disease. iPSCs are developed by harvesting adult somatic cells from the patient’s own tissues [54]. These somatic cells are reprogrammed to a pluripotent state where they can be expanded and differentiated into different retinal cell types including cones, rods, and retinal ganglion cells [55]. Since iPSCs are derived from the same patient there is a very low risk of rejection and ethical issues. Nevertheless, they can still harbor the diseased genes and develop tumors. CRISPR/Cas9 genome editing can be used to correct the mutations in the iPSCs before differentiating them into retinal cells [56].

Apart from direct injection of cell suspension, scientists have also tried bioscaffolds and organoid-derived retina-like cell sheets. Singh et al. (2018) developed a biodegradable porous scaffold composed of gelatin, chondroitin sulfate and hyaluronic acids (GCH) and seed human embryonic stem cells (hESCs) onto the GCH scaffold (Fig. 2B) [52]. They differentiated hESCs into retinal progenitor cells (RPCs) and implanted the differentiated cell sheets (including a variety of retina cell types such as photoreceptors, ganglion cells, and RPE cells, etc.) into the subretinal space of an AMD mouse model. This bioscaffold caused minimal immune reaction after implantation and survived for at least 12 weeks. Although they observed some cells migrated from the scaffold into the inner layer of retina, whether the migrated cells integrated functionally with the host retina is yet to be determined. Assawachananont et al. (2014) developed a 3D retinal tissue from embryonic stem cells and induced pluripotent stem cells (ESCs/iPSCs) [53]. They observed different integration and synaptic patterns after transplantation, and in the cell integration group, the 3D retinal tissue developed structured outer nuclear layer with complete inner and out segments (Fig. 2C).Although they proved the feasibility of retina-like cell sheet transplantation, they did not find the deterministic factors between different integration patterns. Despite the advances in direct cell transplantation with different cell sources and delivery methods (bioscaffolds), these approaches have not been able to significantly improve vision, long term cell survival, nor functionally integrate into the host retina [35], which requires future investigations for better translation into human trials.

### Tissue-engineered approaches for in vitro modeling of AMD

Several in vitro models have been developed to study AMD mechanisms and identify potential therapeutics. They are divided into two major groups: two-dimensional (2D) models and three-dimensional (3D) models. 2D models can be used for researchers to study not only the single cell behaviors, but also cell–cell interactions using conditioned media and transwells [57, 58]. 2D models can mimic some aspects of the RPE and Bruch’s membrane, because these tissues exhibit a layered structure in vivo, which can be modeled in 2D culture system like transwells. Meanwhile, 2D cell culture models have also shown the drusen formation [59, 60]. However, 2D models could not contain photoreceptors (PR) and choriocapillaris structures, which are not easy to be recapitulated in 2D planar. The 3D models can take advantage of bio-scaffolds, organoids, and eye explants to coculture different cell types in AMD, including RPE, PR, endothelial cells, etc. [61, 62]. Moreover, with the help of microfluidic devices, fluid (aqueous humor) perfusion and mechanical force (blinking motion) can be introduced into 3D models, which may lead to a better biomimetic model for the AMD [63, 64].

Regarding cell sources, both cell lines and primary cells are available for modeling retina [65]. Cell lines are particularly suited for rapid and efficient screening of drug candidates for their toxicity and biocompatibility. Different cell lines that have been used for retinal research include the ARPE-19, RPE-1, D407 (RPE cell type), human WERI-RB1, Y79 retinoblastoma derived cell lines, 661 W (photoreceptor cell type), RGC-5 (ganglion cell type), and MIO-M1(glial Müller cell type) among others [66–68]. Although cell lines are widely accessible and easily cultured, up to 20% of currently used cell lines are contaminated or erroneously categorized, thus, validation of its origins and characteristic properties are of paramount importance prior to initiating work with them [69]. Meanwhile, the RPE cell lines were usually isolated from whole eyecups, which means all macular specific characteristics could be lost in those cell lines. Unlike cell lines, primary cells need to be harvested from humans or animals making them less available than cell lines [70]. However, they may better emulate the in vivo scenario than immortalized cell lines. Primary cell cultures used in retinal research include primary RPE cells, primary photoreceptors cells, primary retinal ganglion cells, primary microglia, and primary Müller cells [71].

### 2D tissue-engineered models for AMD study

The simplest 2D model would be culturing retinal cells on plastic dishes. For example, Johnson et al. (2011) developed an AMD model in 2D cell culture. They seeded primary human RPE cells onto a laminin-coated porous supports and cultured with “Miller medium” to trigger AMD pathogenesis. They have observed various pathological aspects of AMD, including drusen-like deposition, activation of the complement system, and deposition of terminal complement complexes in their model. This model highly recapitulates the AMD characteristics [59].

In 2D cultures, cell–cell interactions in AMD were studied with the help of the conditioned media or transwell. Incubating one cell type with the conditioned medium derived from another cell type enabled to study interactions between two cell types in 2D condition. For example, Nebel et al. (2017) incubated ARPE-19 cells, an RPE cell line, with human microglial cell conditioned media [11]. When microglia cells were activated by lipopolysaccharide (LPS) or Leu-Leu-O-Me (LLOMe), the resulting microglial cell conditioned media induced ARPE-19 cells be disorganized and accumulate lipid deposits, which indicates the role of microglia in RPE inflammation during AMD progression. However, their results have not been verified in other primary RPE cell lines or in other in vivo models. In another study, Leclaire et al. (2019) incubated microglia cells with lipofuscin (LP), a waste material that accumulated in human RPE in AMD [57]. They found that microglia phagocytosed LP and increased the expression of proinflammatory cytokines and VEGF, leading to inflammatory reaction and angiogenesis in AMD, respectively.

Using transwells, researchers could achieve coculture of two cell types and enable their direct communication. Meanwhile, RPE cells can form a polarized and functional monolayer on transwell [72]. For example, Ma et al. (2009) used transwell to coculture activated retinal microglia and RPE cells and found RPE cells disorganized and upregulation of pro-inflammatory and pro-antigenic cytokines, which were verified in their mouse models [58]. Transwell was also used in studying RPE interactions with endothelial cells in the choriocapillaris. De Cilla et al. (2020) cocultured ARPE-19 cells with human umbilical vein endothelial cells (HUVECs) on transwell and studied the effect of anti-VEGF drugs on nitric oxide (NO) release from the RPE cells, mitochondrial function, and oxidative status in the RPE cells [12]. They found that when treated with anti-VEGF drugs, HUVECs released paracrine factors to modulate RPE responses, which can normalize NO expression and mitochondrial membrane potentials. In another study, Jeong et al. (2018) cocultured HUVECs and ARPE-19 cells on transwells, found that when cocultured with ARPE-19 expressing TGF-β2, HUVECs decreased expression of vascular endothelial growth factor receptor 2 (VEGFR-2), a receptor of VEGF-A. However, VEGFR-2 did not decrease in HUVECs when TGF-β2 antibodies were added in the system, which suggests that TGF-β2 from ARPE-19 cells plays a role in inhibiting angiogenic responses in HUVECs [73].

### 3D tissue-engineered models for AMD study

**Fig. 3:**
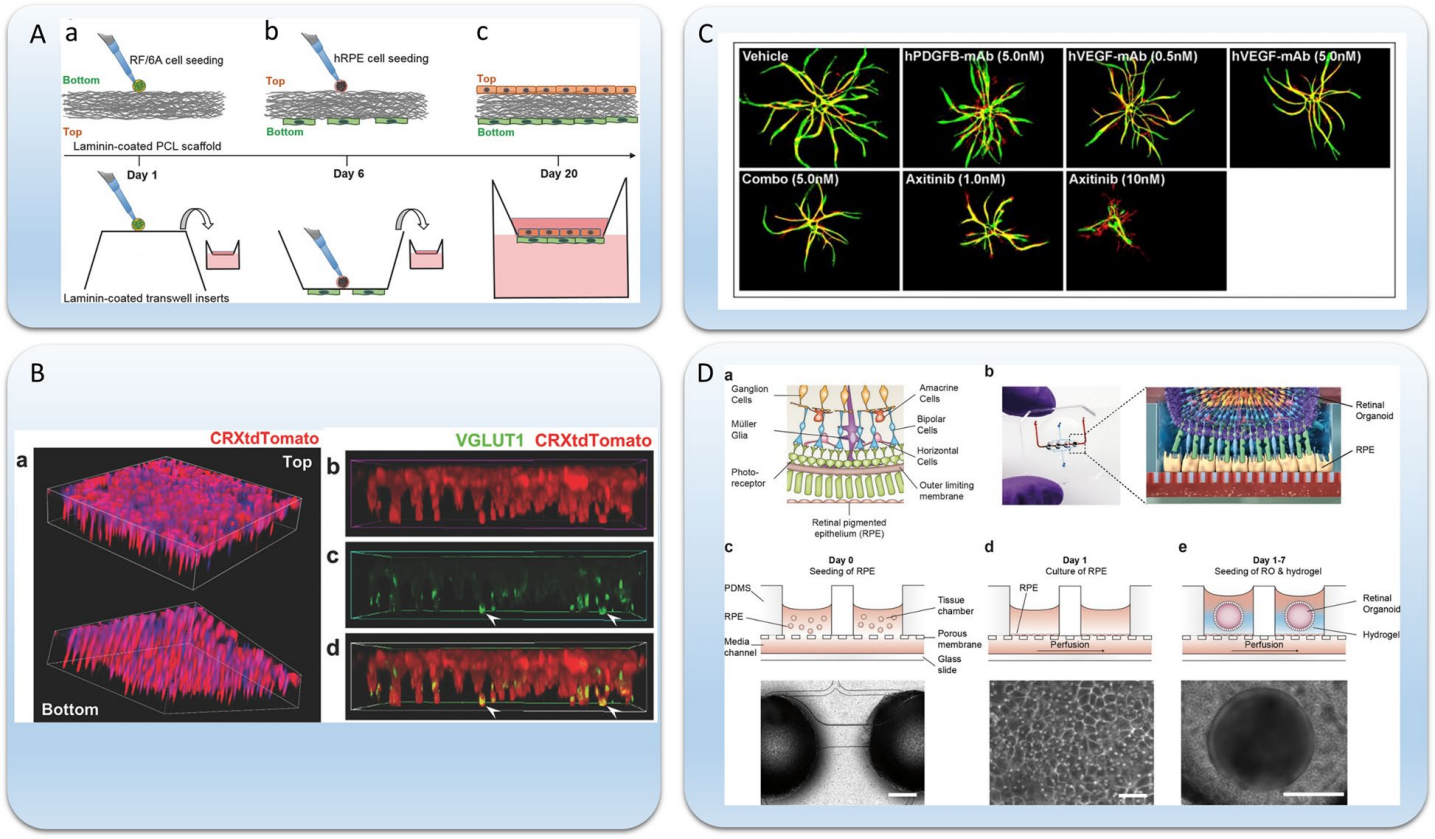
Tissue-Engineered Approaches for In Vitro Modeling of AMD. **A** A 2D coculture model of human RPE cells and primate choroidal endothelial cells (RF/6A) based on Transwell. (a) RF/6A cells were seeded on the bottom side of the laminin-coated PCL scaffolds and transwell inserts and (b) then cocultured with RPE on the opposite (top) side from day 6 before samples were collected and (c) fixed for further analysis at day 20 [62]. **B** PRs become polarized when seeded into the micro-structured scaffold. (a) 3D top and bottom views of the scaffold with seeded PRs reveal processes extending through the microchannels. 3D renderings are 142 µm × 142 µm × 28 µm. (b-d) 3D side view of the seeded cells shows that PR axons are primed for synapse formation via the expression of VGLUT1 on PR axon terminals (arrowheads). 3D image is 100 µm × 55 µm × 23 µm. [75]. **C** Neovascularization assay in 3D hydrogel which shows the effects of anti-hVEGF monoclonal antibodies (mAb), anti-hPDGF-B mAb, its combination and axitinib (a tyrosine kinase inhibitor). The sprouts in green are endothelial cells and sprouts in red are pericytes had similar architecture [76]. **D** Microfluidic retina-on-a-chip model. (a) Schematic representation of the human retinal composition and cell types in vivo. (b) Photo (left) of the retina-on-a-chip and (right) representation of the photoreceptor and RPE interaction. (c) RPE cells are seeded into the device, (d) forming a densely packed monolayer after 24 h of culture. (e) Retinal organoids (ROs) and the hyaluronic acid-based hydrogel are directly loaded from the top into the well and onto the RPE [63]. Scale bars: (**D**) c, 500 µm; d, 80 µm; e, 400 µm. Figures were adapted with permission from [62, 63, 75, 76]

Cell behaviors and cell–cell interactions in AMD could be studied in 2D by using plastic dishes, transwells, and conditioned media. However, 2D models often lack proper cell-extracellular matrix (ECM) interactions, cell responses to realistic 3D environments, and physical forces such as mechanical tension and fluid flow. One of the important extracellular matrixes (ECM) in AMD is Bruch’s membrane, which is a thin, semi-permeable, fibrous membrane separating the RPE and the choroid. As forementioned, Bruch’s membrane consists of three layers: inner collagen layer, elastic layer, and outer collagen layer (Fig. 1B). Bruch’s membrane is a necessary structure for the transportation of nutrients, oxygen, and metabolic waste between the retina and choroid, which is crucial for drusen clearance and RPE-endothelium interaction [74]. Until now, a variety of materials have been used for building a Bruch’s membrane model, including natural hydrogels (e.g., alginate, collagen, gelatin, hyaluronic acid) and synthetic materials (e.g., poly(lactic-co-glycolic acid) (PLGA), polylactic acid (PLA), polydimethylsiloxane (PDMS)). Meanwhile, multiple fabrication methods, including electrospinning, casting, and surface coating, have been applied for modeling a Bruch’s membrane [61]. Although numerous materials and fabricating methods have been studied, fabricating a multilayer structure in Bruch’s membrane is still challenging. Shokoohmand et al. (2017) developed an electrospun polycaprolactone (PCL)-gelatin scaffold as a model of Bruch’s membrane [62] (Fig. 3A). They cultured human RPE cells (hRPE) and primate choroidal endothelial cells (RF-6A) on two sides of the scaffold. Compared to the transwell (~ 0.4 μm pore size), PCL-gelatin electrospun scaffold had a much larger porous size (~ 10 μm), which facilitated cell interaction and migration. However, the thickness of the scaffold was around 100 μm, which is over 20 times thicker than the Bruch’s membrane (~ 4 μm). Based on this bio-scaffold, they observed infiltration of both hRPE and RF-6A cells, which is a critical step of neovascularization in AMD development. They also found upregulation of VEGF and pigment epithelium-derived factor (PEDF) in their coculture, compared with monoculture, showing the importance of multicellularity in studying wet AMD.

While the pro-inflammatory and pro-angiogenic lipids in drusen can lead to AMD progression, the mechanical tension in RPE tissues induced by drusen may be an alternative disease mechanism [77, 78]. 3D culture is useful to study physical forces in the co-culture. To study the mechano-sensitivity of RPE, a bio-scaffold for mimicking Bruch’s membrane was combined with microfluidics to induce deformation and stress. Farjood et al. (2018) developed two different devices for studying acute and chronic mechanical stress in RPE [79]. In their chronic mechanical stress (CMS) device, they used a thin PDMS membrane as the substrate for RPE culture, and a syringe pump to apply high levels of continuous mechanical stress. In the acute mechanical stress (AMS) device, they use a membrane from transwell for RPE culturing and use pipet tips to push against the transwell membrane to induce stress in a short period of time. They found that in both CMS and AMS devices, mechanical stress upregulated VEGF expression in RPE, which proved the role of mechanical stress in initiation and progression in neovascularization.

Another key structure in AMD is the retina, especially the photoreceptors. The loss of photoreceptors owing to drusen accumulation and neovascularization directly leads to vision loss and blindness in AMD. Hence, it is believed that rebuilding the polarized structure of photoreceptor cells in vitro is promising for studying neuroprotection and neurodegeneration mechanisms in AMD and developing cell therapies. To achieve this, micropillar scaffolds were used for guiding photoreceptor cells and other retina cell types to form a polarized structure in vitro. McUsic et al. (2012) developed a PLGA, poly(lactic-co-glycolic acid) micropillar scaffold for reconstructing organized retina tissue through lithography and casting [80]. By seeding dissociated retina cells isolated from newborn mouse into the microchannel scaffold, they achieved high viability of cells and lamination between inner retina cells (amacrine and ganglion cells) and outer retina cells (bipolar cells and photoreceptor cells), which was confirmed by anti-Pax6 and anti-Otx2 staining. They also combined the microchannel model of retina cells with a mouse RPE tissue ex vivo; and observed rods extending rhodopsin-positive processes toward RPE. Finally, they tested human embryonic stem cell (hESC) derived photoreceptors on the microchannel scaffolds and found similar characteristics to that were observed in their mouse retina model. With the advancement of microfabrication techniques, Jung et al. (2018) developed an improved microstructure scaffold by wafer etching and PDMS casting [75] (Fig. 3B). By combining isotropic ion etching and anisotropic etching, they fabricated a scaffold with “wine glass” microstructure with a cone-shaped head and a rod-shaped tail. The cone head could capture the photoreceptor cell body and the rod tail could guild the basal axon extension. They differentiated photoreceptor cells from human pluripotent stem cells (hPSCs) and seeded them into the microchannel scaffold. They found the optimal photoreceptor behavior on PDMS scaffold with plasma treatment and laminin coating. Actin staining also showed axon extension in the rod tail of the microchannel scaffold. Interestingly, the differentiation and maturation continued after photoreceptors were seeded. While PDMS is not biodegradable, they also developed a poly(glycerol-sebacate) (PGS) based microchannel scaffold, which is biodegradable and showed the similar photoreceptor behavior. 

Three-dimensional (3D) printing technologies are used for building a photoreceptor model. Wang et al. (2020) demonstrated 3D printing using functionalized hyaluronic acid (HA) to improve photoreceptor differentiation from retinal progenitor cells (RPCs) [51]. They functionalized HA with methacrylation through a glycidyl-hydroxyl reaction to get a photopolymerizable hydrogel (HA-GM). Then, they 3D printed a dual-layer structure for the coculture of retinal progenitor epithelial cells (RPEs) and retinal progenitor cells (RPCs) for RPC differentiation to photoreceptor cells. They found high RPEs viability in HA-GM that has stiffness close to native retina. Despite the high viability, their RPE cells didn’t form a monolayer in HA-GM hydrogel. After two weeks of coculture, they observed significantly increased expression of rhodopsin (a marker for rod photoreceptors) and M-opsin (a marker for cone photoreceptors), which indicates that 3D coculture with RPEs improved RPC differentiation to photoreceptor cells. Although this is the first study that applied 3D bioprinting in AMD study, their model requires more thorough biological validations to be convincing, including RPE and PR gene expression and morphology studies.

Vascular structure is also crucial in AMD, especially in wet AMD. Hence, there have been 3D models to evaluate vascular and angiogenesis activity of the choriocapillaris. Giddabasappa et al. (2016) used a 3D coculture sprouting essay to evaluate the angiogenesis activity of human retinal microvascular endothelial cells (HRMVECs) when treated with axitinib, a small molecular tyrosine kinase inhibitor [76] (Fig. 3C). In the 3D sprouting essay, HRMVECs were first coated on microcarrier beads, then HRMVEC-beads and human mesenchymal stem cells (hMSCs) are embedded in the 3D fibrin gel. The coculture gel was cultured for 2 weeks to observe HRMVEC sporting. In their setting, they found axitinib inhibited vascular sprouting with a higher efficiency than anti-VEGF or anti-PDGF-B antibodies.

Because of the impact of shear stress to endothelium, fluid perfusion is a necessary factor to build up a biomimetic vascular model. Hence, microfluidic devices are widely used for choriocapillaris models. Chen et al. (2017) developed a microfluidic device to coculture ARPE-19 and HUVECs to study their cell–cell interactions [64]. Based on soft-lithography, they built up a micro perfusion device and seeded ARPE-19 and HUVECs on two sides of a porous membrane made of PDMS. In the device, ARPE-19 cells expressed a tight junction protein, ZO-1 and formed the intact monolayer. They used a low glucose or a hypoxia condition to trigger AMD and found upregulation of VEGF in RPE in the low glucose or a hypoxia condition. Interestingly, because the PDMS membrane (6.5 μm) is much thinner than other previously developed scaffold (~ 100 μm), they observed RPE detachment and HUVEC invasion through the membrane, which is consistent to pathological macular neovascularization (MNV) observed in wet AMD. This study showed that MNV can be affected by glucose and oxygen levels.

Although most 3D retina models recaptured certain cell types in AMD, they could not integrate all the cell types in retina in their models. Hence, the development of retinal organoids represents a leap forward in a more realistic disease model. Organoid cultures are 3D structures of self-organizing organ specific cells derived from stem cells [27]. Retinal organoids typically consist of spheres that contain cellular layers of multiple retinal cell types. By retaining the complex connectivity of non-neuronal cells and neuronal retinal cells, retinal organoids can emulate in vivo condition. Dynamics of cell–cell interactions, immune responses and degenerative pathways may also be studied. A recent breakthrough was achieved by Cowan et al. where human retinal organoids capable of processing visual information were produced from iPSCs [81]. These human retinal organoids had multiple nuclear and synaptic layers and functional synapses. Furthermore, they created the first ever single cell resolution gene expression atlas of the human retina and choroid from extra fresh (less than 5 min post-mortem time) post-mortem human retinas and compared their organoids to this atlas.

Although retina organoids integrate different retina cell types, they lack vascularization and cannot distinguish retina-RPE interactions. However, these drawbacks can be overcome by using 3D microfluidic systems. For example, Achberger et al. (2019) developed retina-on-a-chip model, which combined human iPSC-derived retinal organoids with human iPSC-derived RPE, where the inner and outer segments-like structures of the photoreceptors were formed and preserved [63] (Fig. 3D). Furthermore, interactions between the RPE and the photoreceptors, and vascular-like perfusion were recreated in their system. Based on the preserved histotypic context in organ cultures, the molecular and cellular pathways of cell–cell interactions can be better studied. However, their claim on inner outer segment formation based on the ROM1 staining and scanning electron microscopy (SEM) images is not fully convincing. While ROM1 is a marker for the outer segment, it has been shown to express differently in 2D and 3D cell culture systems without outer segment formation [82, 83], hence ROM1 expression doesn’t necessarily prove the outer segment formation. Furthermore, their SEM image didn’t show the typical layered morphology of the outer segment. Hence, the interactions of retina organoids and RPE cells are still in need for further studies.

Ex vivo studies using eye tissues in 3D have been performed. Transversal retinal slice preparations of approximately 200 µm in thickness have been imaged under a single or two-photon microscope without doing tissue fixation. Physiological and pathophysiological observations of individual retinal cells under high spatiotemporal resolution were obtained in real-time. A major disadvantage in this method was that retinal slices survive for only a few hours after preparation, which limiting long-term observations and biological treatment [26]. Neuroretinal explants with or without RPE, RPE and choroidal explants, and whole eye explants have all been described [84]. Current neuroretinal explant models may mimic the in vivo situation more realistically when compared to cell cultures. Multiple cell types are present in these models which permits observation of in vivo dynamics of degeneration pathways, microglial responses and dynamics of cell development and interaction. There are several limitations for their use of ex vivo tissues in AMD research, which include the absence of drusen, lack of vascular perfusion, and the relatively short lifespan of the explant when compared to the real disease progress. Moreover, the ex vivo explants have been derived from rodents that lack a macula. An alternative would be to consider human-derived retinal organoid cultures: a challenge would be the availability of human eyes plus the legal and ethical ramifications [69].

### Future perspectives

In the past decade, there has been a major push to eliminate animal testing. This means that the need for in vitro models is a topic that will grow in relevance as we move forward. Compared with traditional 2D culture or co-culture models, 3D AMD models have shown significant advances, including better biomimicking, simulating cell-ECM interactions, demonstrating RPE and endothelium migration, and drusen formation, etc. Hence, we believe 3D models will be a promising approach for AMD modelling in the future. Meanwhile, in most 3D models that we mentioned above, only limited cell types were considered. For example, Shokoohmand et al. (2017) considered RPE and HUVEC migration but didn’t include photoreceptors; McUsic et al. (2012) provided a novel microchannel scaffold for photoreceptors and RPE but didn’t include any vascular structure. A 3D model that can integrate RPE, vascular, and photoreceptors is in great demand. Considering that, we think retinal organoids, retina-on-a-chip and organ culture models with appropriate vascularization will be able to revolutionize our understanding of complex retinal diseases such as AMD in the future. The development of a 3D culture that reconstitutes human ocular structure to permit controlled experiments investigating drusen formation, deposition, and development of macular atrophy or macular neovascularization in AMD will push the field forward. Furthermore, most choriocapillaris models seeded endothelial cells onto 2D surface with no flow. By combining the AMD model in a 3D microfluidic device would achieve a 3D vascular model with shear stress, which can better mimic the in vivo condition in wet AMD. 

## Data Availability

Not applicable.

## Abbreviations

AMD: Age-related macular degeneration
NVU: Neovascular unit
CC: Choriocapillaris
RPE: Retinal pigment epithelium
BrM: Bruch’s membrane
SDD: Subretinal drusenoid deposits
MNV: Macular neovascularization
MA: Macular atrophy
VEGF: Vascular endothelial growth factor
VEGFR-2: Vascular endothelial growth factor receptor 2
iPSCs: Induced pluripotent stem cells
PR: Photoreceptors
LPS: Lipopolysaccharide
LLOMe: Leu-Leu-O-Me
LP: Lipofuscin
HUVECs: Human umbilical vein endothelial cell
NO: Nitric oxide
ECM: Extracellular matrix
PLGA: Poly(lactic-co-glycolic acid)
PLA: Polylactic acid
PlGF: Placental growth factor
PEDF: Pigment epithelium-derived factor
PDGF-BB: Platelet derived growth factor BB
PDMS: Polydimethylsiloxane
PCL: Polycaprolactone
hRPE: Human RPE cells
CMS: Chronic mechanical stress
AMS: Acute mechanical stress
hESC: Human embryonic stem cell
hPSC: Human pluripotent stem cell
iPSC: Induce pluripotent stem cell
HA-GM: Methacrylated hyaluronic acid
ROs: Retinal organoids
PGS: Poly(glycerol-sebacate)
RPCs: Retinal progenitor cells
HA: Hyaluronic acid
HRMVEC: Human retinal microvascular endothelial cell
hMSC: Human mesenchymal stem cell
GCH: Gelatin, chondroitin sulfate and hyaluronic acids
DICs: Differential interference contrasts
mAb: Monoclonal antibodies
BSS: Buffered salt solution
